# Seventy-Five Cases of Solid Tumours Treated by a Modified Quadruple Chemotherapy Regime

**DOI:** 10.1038/bjc.1971.59

**Published:** 1971-09

**Authors:** I. W. F. Hanham, K. A. Newton, G. Westbury

## Abstract

**Images:**


					
462

SEVENTY-FIVE CASES OF SOLID TUMOURS TREATED BY A

MODIFlED QUADRUPLE CHEMOTHERAPY REGIME

1. W. F. HANHAM, K. A. NEWTON AND G. WESTBURY

From the We8tmin8ter Ho8pital, London, S.W. I

Received for publication April 7, 1971

SUMMARY.-Seventy-five cases of malignant solid tumours treated by a
quadruple chemotherapy regime are described. These tumours originated
in the breast, head and neck, bronchus, genital tract, cutaneous melanoma, soft
tissue and gastro-intestinal tract. All 14 patients with breast carcinoma
underwent remission and in 6 this was complete. Significant remissions were
seen in gastro-intestinal and head and neck malignancies, and also in the soft
tissue group. A short response was noted in 6 of 14 cases of bronchial carci-
noma. Malignant melanoma, testicular, ovarian and cervical carcinomata
failed to respond.

In all, 40 of 75 patients underwent objective remission.

RESULTS of treatment of solid tumours (as distinct from reticuloses and leukae-
mias) by individual chemotherapeutic agents have been disappointing, although
short remissions in 50% of breast cancer cases have been observed with cyclo-
phosphamide (Kunkler et al., 1968) and 30% with 5-fluorouracil (Heidelberger
and Ansfield, 1963). It was at one time hoped that concentration of dose by
intra-arterial techniques would improve the response rate for localised tumours,
but this method has fallen into disfavour at this Centre owing to frequent relapses
and a disturbing incidence of complications. The position of intra-arterial
chemotherapy, certainly in head and neck cancer, has further been weakened by
the results of intermittent high dose intravenous injections of Methotrexate,
which have given a 57% remission rate in one series (Leone et al., 1968).

In an effort to improve the generally disappointing results associated with single
cytotoxic agents, intravenous injection of a combination of the 4 cytotoxic agents,
cyclophosphamide, Methotrexate, Vincristine and 5-fluorouracil, originally advo-
cated by Constanzi and Coltman (1969) has been selected, though in a reduced
dosage (Table 1).

EXPLANATION OF PLATES

FIG. I.-Nodular carcinoma en cuirasse before (A and B) and 3 months after (C and D)

chemotherapy.

FIG. 2.-Breast carcinoma. Widespread diffuse opacities both lung fields, before (A) and

2 months after (B) chemotherapy.

FIG. 3.-Careinoma of bronchus with cutaneous metastases before (A) and 1 month after (B)

chemotherapy, showing partial response.

FIG. 4.-Cutaneous deposits from alveolar rhabdomyosarcoma before (A) and 2 months after

(B) chemotherapy. Mediastinal deposits before (C) and 2 raonths after (D) chemotherapy.

m
6

0
1:

t:q

r--4

. !QiMo.
,x I]

IA            . ".41

. ..;kp
FA

Q

F:
I

U

P4
0

z              .   .
9
0
?-z

?g
&O
?-4
1-4
0.4
pi
m

1.0

Q
00

4)

9
0
0

4a

6

03

T---i 1

03

N

Vol. XXV, No. 3.

BRITISH JOUR-NAL OF CAlITCER.

2A

2B

Hanham, Newton and Westbury

BRITISH JOURNAL OF CANCER.

Vol. XXV, No. 3.

3A

3B

Hanham, Newton and Westbury

BRITISH JOURNAL OF CANCER.

Vol. XXV, No. 2.

4A

k.. .

L,

4C

4D

Hanham, Newton and Westbury

MODIFIED QUADRUPLE CHEMOTHERAPY REGIME

463

TABLF, I.-Quadruple Chemotherapy

Constanzi and Coltman, 1969,                      Modification of dosage in the adult

Cancer, 23, 589                                 suggested by the authors

300 mg.-2 doses days 1 and 5       Cyclophosphamide    2-300 mg.-2 doses days 1 and 5
0 - 5 mg./kg./day-2 doses days     Methotrexate        0 - 25 mg./kg./day-2 doses days

I and 4                                                1 and 4

0 - 025 mg./kg./day-2 doses days   Vincristine         0-015mg./kg./day-2dosesdays

2 and 5                                                2 and 5

10 mg./kg./day, daily              5-Fluorouracil      7 - 5 mg./kg./day, daily for 5 days

Constanzi and Coltman (1969) suggested that the interval between the first
and second course should be 2 weeks, and thereafter 4 weeks. This regime was
followed in the early cases, but we now find that'a 4-weekly interval throughout
is effective. The modified schedule was adopted because severe toxicity was
seen in early cases treated according to the original protocol. Further reduction
has been made in individual cases for any of the following reasons:

1. Old age.

2. Previous chemotherapy or radiotherapy.
3. Widespread bone marrow involvement.
4. General ill health.

In the series of Constanzi and Coltman (1969) treatment was continued for an
overall maximum period of 6 months. In the present series patients have been
under treatment for periods up to 18 months.

Clinical material

Seventy-five patients with solid tumours have been treated: Table II indicates

TABLEII.-Types of Tumour Treated and Degree of Response Achieved

Objective remissions

Complete Partial Failure  Total
Breast carcinoma           6        8       0       14
Head and neck carcinoma    1        7       2       10
Bronchial carcinoma        0        8       6       14
Ovarian carcinoma          0        0       4        4
Testicular carcinoma       0        0       4        4
Cervical carcinoma         0        3       0        3
Malignant melanoma         0        0      10       10
Soft tissue sarcoma        0        5       1        6
Various

Nephroblastoma           0        1       1        2
Hepatoma                 0        0       1        1
Stomach carcinoma.        I       I       0        2
Large bowel carcinoma     I       1       1        3
Bone carcinoma           0        0       2        2
Total                      9       34      32       75

the type of tumour and the degree of response achieved. Objective response was
graded as:

1. Complete
2. Partial
3. Failure

464

I. W. F. HANHAM, K. A. NEWTON AND G. WESTBURY

The Karnofsky scale, Table III (Karnofsky and Burchenal, 1948) to indicate
subjective response and improvement in the general condition of the patient.

TABLE III.-Karnofsky's Rating for Chemotherapy Response

Normal                                           100
Minor signs or symptoms                           90
Normal activity with effort                       80
Unable to carry on normal activity, but cares for self  70
Requires occasional assistance with personal needs  60
Requires considerable assistance and medical care  50
Disabled                                          40
Severely disabled and hospitalized                30
Very sick: active supportive treatment necessary  20
Moribund                                          10
Death                                              0

Complications

Thirty-six cases were treated without side effects. In the remainder the most
frequent was evidence of toxicity, leucopenia (2000 white cells or less), followed by
alopecia, nausea and vomiting, stomatitis and peripheral neuropathy.

Bone marrow depression (16 cases) was no more frequent or profound than with
standard courses of, e.g. Methotrexate, 5-fluorouracil or cyclophosphamide used
singly.

Alopecia occurred in 15 cases, mostly in the early part of the series. It is
possible that this incidence would have been greater had it not been for a scalp
tourniquet applied during and for 5 minutes after injection.

In 3 patients with peripheral neuropathy Vinblastine was substituted for
Vincristine in view of the known neurotoxic effect of the latter compound.

Three patients failed to complete their treatment because of side effects.

RESULTS

Breast Carcinoma (Table IV)

These were patients with advanced and uncontrolled disease, which had
previously been treated by hormones or adrenalectomy, and/or 5-fluorouracil
or cyclophosphamide by injection. All cases showed some response; in 6 out of
14 this was complete and maintained for more than 6 months.

Subjective improvement was manifested by relief of bone pain and improvement
in general well-being. Objective remission was observed in chest wall recurrence
(Fig. IA, B, C, D), in liver deposits and in pulmonary metastases.

In I patient lung function studies were carried out before and after 2 courses of
chemotherapy. They showed an increase in total lung and initial residual
capacity and in maximum expiratory flow rate (Table V, Fig. 2A, B). Chest
X-ray confirmed some clearing of disease.

Two patients were considered too ill for endocrine ablative procedure, but
following good objective and subjective response to quadruple chemotherapy,
successfully underwent bilateral adrenalectomy and oophorectomy. Remission
continued without further chemotherapy in both cases.

Four patients with carcinoma en cuirasse were treated; I showed complete
response and 3 partial response. In our experience this type of disease has proved
unresponsive to single agent chemotherapy.

465

MODIFIED QUADRUPLE CHEMOTHERAPY REGIME

TABLE A'

Predicted           2 months
normal    Before     after
a. Spiroinetry (litres)

P.E.F.R.                 417       165       185

F.V.C.                     2-44      1-27      1-43
F.E.V.(l sec.)             2-09    0-84        1.09
F.E.V.                    86%       66%       76%
F.V.c.

AI.M.E.F.R. L/See.         4.99      0-56      0-84
M.M.I.F.R. L/See.          3-74      1-43      2-16
M.M.E.F.R. Ratio           0-8       0-39      0-38
M MT. I. R.

1). Lung volvittes (litres)

Slow V.C.                  2-44      1-27      1-43
F.R.C.                     2-26      1-7       1-64
T.L.C.                     4-05      2-53      2-47
R.V.%                     35%      49-9%     41-1%
T.L.C.

Head and neck carcinoma (Table V1)

The palliation of uncontrolled head and neck cancer presents a challenging
problem to the chemotherapist. Intra-arterial methods have fallen from favour
in this Centre, and, as already mentioned, single intravenous weekly injections of
Methotrexate have produced comparable results with far less morbidity. One of
us (I.H.) has observed objective response in 50% of patients treated by intra-
venous Methotrexate, but palliation was generally short-lived, seldom exceeding
3 months.

Quadruple chemoti-ierapy has been used mainly in those cases showing failure
of control with, or relapse following intravenous Methotrexate. Further remission
has been achieved in 6 of 10 patients, lasting up to 6 months in I case.

In view of these findings we suggest that quadruple chemotherapy should

replace the use of a single agent in this gro-tip of cases.

zn

Bronchial carcinoma (Table VII)

Fourteen cases have been treated. None showed complete regression of
disease, but in 8 patients there was a partial, short-lived, response (maximum
duration 3 months). Rapid, though transient, regression of cutaneous metastases
was noted in 3 patients (Fig. 3A, B). One patient with superior vena cava
obstruction, which had relapsed after radiotherapy, was treated (by injection into
the veins of the uiiinvolved lower limbs) with rapid resolution of symptoms.

Our experience suggests that the results may not be better than those following
single weekly intravenous injections of cyclophosphamide, though admittedly
this series is small.

Genital cai-cinoma (Table VIII)

Four testicular and 4 ovariaii carcinomas were treated, without effect.
Wiltshaw (1965) claimed 43% remission for at least 2 months in ovarian cancer
using chlorambucil; Bateman (1962) reported a similar remission rate with
ThioTepa, and Burns et al. (1969) claimed 50% response in carcinoma of the ovary
treated with phenylalanine mustard. The poor response to quadruple chemo-
therapy is therefore surprising.

38

466

.5

as

P4

4-1
0

4-4
-0

-.4
2
6
-4

0

. 4

. ILIQ

e.)

e

111-Z

OD

8
pq

'4Q?

Z3
Q)

9

14)

v
eIt
Q
Pr4

I

?0:
?-q

rA

4-4              00

0

4D

0

ok

0
k

0

0                k

o             0

P-4   z

0
0

4T

2
0
m

4)  m             m        114        to        l-

b(

.,?    CZ)        -ld4   L-i           10        1141

4-'?  .
0

(D

i * 4

P.,

4     ;4     6
?A    ?      C4

0

c ag)

4Z

0 94

0.2

,O C?4
10 0 Ca

co

?,,A4 ?-4

0 0
CD

(D

0
-4

4a     0

0      0

O      O            O        Cl

O

o            0 O                0
00           00    00

(D                              a)
74j

-6a

P-1   N                      P.,

C)    C)

0

14    Q         0

CB

0

4D 4

C.) 4.3 0

0

o

0        4)
00       m

m
as    a)

1.0      g 4
m        m 0

...4 C)  -4  C)
4a      4a

0     4 0
4 0        0
0        0
m        m

467

C*

'o                        0      0    0 as              0

CH     CH    ts-i 0           44

o

04                                         ?

00 00

m         Go        m                                W

0    C> 0      0

00      m    00               m

C>        0         C>        C>      0    0    0    0

00                  00        10      W    10   m    00     m

CB

0                                0         0      0

CB
0    CB

0
0                      0

Cs

0 0
-&a k

0 I'D' 0-

-q                                                   0
0

o

0            0

o

0         0            o 'as

0 0    PT         4        ,  0, ?     ,

0                 W                0         o    o o    'O

0

0
oto                   16   H

x    16      x

0

3)               0          ID  C)

bo                               =

-4

P41                   -4a       4a   4-,)

0

0                   0                 0         0    0      0

C>   to   10  lqdq     0
10           W    L-  t-      t-

468

Q (D

O -43

bo

04    bo                      0

0           &4 0
o to

4a            .4a

N              0 -.0.         0

C4-10
o

P4
0

>

co

P4                            P4

Mz                      Z        Z

4-D

0 .2                             2

(D                              ti-4 k 0

E                                as 0  N            0  f.

0                             (D 10 -Q       ;4

'+""0                             0     P        o
2                                   .0,4 0   7g

pq

Qo                   0     E.2 4,
>                                r...

0-51 "

0

Pk o                     0        0

?4

4-i

0           0        0        0              0

Cs

co       CB       Ca             Cs

0                             0               0

0 0

co

;T4

co
4)

co                      06       P4

469

14

0

$M4       so                       P4

co

0                 A o

04           04                    04

o                     0            0  bjD                0

r.

NII                       4Q

O                     Q            C>                    O
co

Q                                  Q                     Q

UZ

co

4-'-')

0

U                                     P-1                                P4

co

P4
0                                  0
C)                                 C)

=1                                 0
7=              -4                     tn

z               z                     ?4           z

0

Z
o a)

4a

4D

4a

0  0         0                        4a Cs

0                               (D                       ;64

p

P,                       C)                                       4a

o            o o o              2

F-4 ?oN         94

4a                                                    04

0                               0 0

CO)   8)                 co

co

P4                    P4

0                  0

0

(D 0                  (D 0            0                               (D

0     Cs                                          'Io

0               o                                                        1:14.

4a 0
4a

4      0)           0  0            co                    (D

o                     R,                                              o  C) C)

co                                                                      41)

14

00
co            10

lf?      O                aq
co       m                to

470

I. W. F. HANHAM, K. A. NEWTON AND G. WESTBURY

Three cases of cancer of the cervix were also treated and failed to respond.
Malignant melanoma (Table IX)

Ten cases of malignant melanoma were treated without response, failing to
substantiate the encouraging results suggested by Constanzi and Coltman (1969).
Seven patients survived for more than 6 months, but it is unlikely that quadruple
chemotherapy altered the natural history of their disease. It could be argued that
our modification of the dose regime contributed to these poor results, but our
overall clinical impression is that this drug combination is of little value in malig-
nant melanoma.

80ft tiSSU6 8arconm (Table X)

The value of chemotherapy in soft tissue sarcoma is not generally recognised,
although Wiltshaw (1967) has reported benefit following oral Methotrexate.
Arterial infusion or perfusion may also produce regression in these lesions (Newton,
1967).

We have treated 6 patients, using the quadruple regime. A boy, aged 14,
with metastases from an alveolar rhabdomyosarcoma involving mediastinum,
liver, pancreas and skin, showed complete clinical regression within 2 months of
starting treatment. This patient continued in reasonably good general health
for a further 15 months, but isolated recurrences in the right inguinal region,
pancreas (proven at laparotomy) and mediastinum required local radiotherapy
(Fig. 4). Unfortunately this patient has now died from intestinal haemorrhage,
20 months after starting chemotherapy.

Four other cases continued to show partial response.

One case only, a leiomyosarcoma, failed to show objective response.
Carcinoma of ga8trointestinal tract (Table XI)

Two patients with carcinoma of the stomach and 2 with carcinoma of the colon,
and I with squamous carcinoma of the ano-rectal junction have been treated.
AR showed some response; this was clinically complete in I case of carcinoma of
the stomach who remained in remission for 3 months, eating normally and gaining
weight. Barium meal examination showed reduction in size of the growth.

The patient with metastatic carcinoma of the ano-rectal junction is now in
complete remission, 6 months after commencement of treatment.
Miscellaneou8 (Table XII)

No benefit followed treatment in the remainder of the series, which included
patients with nephroblastoma, hepatoma and bone sarcoma.

DISCUSSION

Seventy-five patients have been treated by a modification of a regime originally
reported by Constanzi and Coltman (1969). This modification was necessary
because of unacceptable toxicity in our earlier cases. Using the modified regime,
treatment now present no undue problems of toxicity, yet still retains a therapeutic
effect. It is practical to treat the majority of cases as out-patients. The most
troublesomesideeffect(especiallyforfemalepatients)isalopecia,20%. Haemato-

471

MODIFIED QUADRUPLE CHEMOTHERAPY REGIME

.4

4    0 ?-,
D
I

a 4-4 0 4a

a 0 PI 0 m m

5    'm 0

C)

14

I

>., 4Z

4    M     (a) C>
a    0 ?-o 00 00
.4   N

D

.4
-4

a -  6     O 0
1    k -   t- co

P-1 0-0
I

9
.1c

-4Q

(t
I"

r-
I

I1?
L4
I            c
0     a)
.9 2

?2    0     ?4

t? pm--
? ) 0
, 9

P--b
.'IQt?
Q
9
2
pq
"IQ?

CO

rs
Q)
9

14)
4Q.

(Z)

pr?

1-

-4    rz -.4           -4 rz      1-4

. '.4 m          aq    " z r-i     " r-4 r-4

?4    z ??4            z           z

O 0 0 (?      O    O 0 O     0 O O
10 00 10 t-   00   *I w co  10 00 t-

O O O 0       O 0 0 O O 0 O
la 00 10 r-   00   m co 10  CD co cm

O
ka

0
10

. P4  ?i

0?  -? ?i C?   F! P4 ?4

:A ?: N   ?-- ?!i -?

(D

M                                  GM

S
4Z                      4

0 0 0 4-4

GD

E-1

N Lo *4        (D      aq aq cq          aq

0     0 0

0 C) S                    40- 0    0 0 0

4-4 4-i                                                         4-4

CB 03                                               CB          as

Po    A '-d

0        o                 4

0.0         0                   A.2

. . C)                          0

(D

(1) -    CD (1)           (E) (D        a)

co co                                                                   10-31
4-i . q -4

C) 4-i 4a                            4.'j         4a        -4a 4a           4a
(D    ?  k       -.4 -6-'a

co       .,.q                       I   85

co

P? P4            P4 Prq N        P4     P4 N   P-4   P4 P4 P-4            P4

ra4
0

Z, Z,4                     z z

4-4

0                              0                0

0 0

CD

0 0

cq 10            aq lfl? eq      C*                  lo t- to            10
bio CO 10           to to 10

a             .   .   .   .

0

0            m ?4

.,4  . .      C) 0  .

4:) p ?o

co           ?? X X

N  t4 P4     P4 ?i P4 ?4

472

1        1

R

;? o

..

0
?! 0

P-Q  In:11

0

.'eQ  ;4

z

41        I

9
w
;Z)
P-Q1w
;44-

1

pq
0.4
Po

E-?

I. W. F. HANHAM, K. A. NEWTON AND G. WESTBURY

to                             bo

0 0

44

-.0           m                          MD

as

-0

0

4Q

o                          ti-4

z         z    z   z       z       z   z  z   z

o 0
P.,

00             cq                  m   m

as

. 4

C)

4)

P4
0
1.4

..!4

Ca

CD
m
0
,--q      --(     Ca

z z z

N      1-4  -4     -4   m         m         N.  Nl   N    -4

0

pi

P4

0

-4a
0
0

Id

10
C)
k
0

x

14
aq

m
-iz

C.)

(E)                     d

14                   es C)

4)       CS         .." m

0        (D         C)

OD         a)

10                   04 9
-4

m         9          0 0

z

4a

04,0  P-4         (D       F-4

0            4D       4D

.1.4                         0                 4a

P'o      4,9z4AZ4                              lm 0 ? 0 m

4..   r,       r, -4z 0
U     4) 0        0

(biO - 'q                              -, 0

9)             t-       .*      to   I 00

,C4    CD       co       co    w     a aq

9

. ab

E ,
4a                                   8

9                                   --b

4)

-6a . &6        P4

co                      w     ?l    .9 P4

44     P4       ?4       6       x   t., ?-?

'k)
Q

MODIFIED QUADRUPLE CHEMOTHERAPY REGIME

473

D

CD

W

1?4

(D

(D 4-D

, O'

0- o

ap

CD
co
w
CO

C4-f

0

?-Ill

C)     0     <Z

r-    t-

O O
km 00

Q
lf?

la

(C)        c         c

1-        t-         t-         I fl? (30

-   1-4     I--(

"F  ?i      ?241              114

MN          C111               -4

A;   ,     I

1:?

4        P4 ?-    r/?

6
06

4)              (1)          (1)                      (1) (1)      (L)

k                            r-4                                                       2
0                            0            9

I                         " 1-4                           I"

. -  . -4    . -

Cs               Ca          Cs           CB          Cs  03       Cs                  'a

Pr.(             PLI         PLI          Pro         44 44        PL,                 PL4

ca          Cs

i           9           0

(L)

Q                       P-4
(2)         0           0
9L4        r.)          C)
0           z           z

--4          C)         (L)

't    ?4           4
(1)
. m

z
C3

-4              m     r.   lf?         -t

eN

ca

CD                                  0?4

m . 4 (D

bo

4a 0

bo

x

Iq

4--J
0

(D

"C'S 4    &6    P?          ?4
P-1 P?    P4    P4          P4

474

(D

04  4    0

s   P4                                           v. P.,

4-.

4.

o

44

0

o  bo                                  "d       0 bio

0.5                        0           0

41

OD                                              00

0        Z        (M    co       C>           m

z
(D
N

4.'.)           C>    O        0           O           O
00'm                                                   01

(kD,,o (= >    O     O        0            C>          O

P4 0   M        00   (M        m           00

4)

>                           4

4a  v                        0

4--)         0       4a
-4a

k     %        0 4a P4

CB

P4       pq                         P.,         P4

4Z-

(D
04
0

0
0

z     z                                ?4

cq    co       00                      aq
0

0

C3

.4

0                          4-4
00                         Cs

C)

0   0                                               C) k

4a

03             P4          P4

4-D          C) 4a      CD

0           (D M

0 Cs m I    -0-            P4

4Q ?   oi                   (D

44 C)                                     A

C)

0

P     P4       P

0 0            6

o o
0            0

0                          0

;>a 0                               0

9)                            -.0 00    I5 00             o

A       VC.,                -04             $4

.0 co     0                                -C.   4-D

a.

P4

P4

C>

72  p.;      P4

475

7;?l (E)

m
44
0
0 0

tv I 'm

16

0 (Cl)) , Z

.-4

la 4a

as

0

"-I 4.'.)

CB m

-40
0

(D

I

I

I

aq   to

co P-1

O O
CD co

C) 0
co to

0 C) C)
'0-'? m oo
0

0 P4

o

0           P4     10
(D

(D

143

4)0

0     (D

M  L-    aq

0

(D
Go
co

pq                   0        -4-D

.,4

4Q

O
O

P-4

0
00

C4.4  44                 44
0                        o

CB                  CB   Cs
0  5 2              0

.0   0              o

0     4
.4z  co

OD   m    C)        0 C) 0

U

7g           o6
P., P4  d    94

k           N
Ca     ? '-d  4)

o   4
C)      Cs  as

..4      0   a)

% w m   o   o   >,.

I g g   0   0   P4

? 0 0   ?     - 4

0

-0

.0 ,, 0     oo

,al.

p 2 -

S

4)            -4     -1

4            w       Cs

.,-4   ..4

t             Q      -Q
0

U             P4

e

-4

-   Cs     :t? co
Cs P4     4.5 -,-Z
16, o      c a C.)
o             0
4       (D      04

$4 0   0

0 45

14 2 > m 'Cs

1.1;3          0      (D
z             N m

to            -d4     P-4

476

4-D

o

3

4Q 04             4--)
0    -4           0

0 bo        o bo i?           o  bo o bo

E.5         ? -E                    EA

4a          4-D

% 8

4                                   4-11

4-D      4--)

0     o

o Z

C)                C     O
00                C*    m

P-4
0

C>       0     O

0

P-4

4a

A

4Q

(2)

4Z

4Z

4a

00

O..      4-

0 0 4a                           ND

0

4a     E

0               0 0

0      C)             0

-4a       ;6.,  4--

b                        4Q1

4-J     C4;4-

P4        ?i      0       9

PL,

o

4Q
0

79                       P?

P4                       ci       w     4

MODIFIED QUADRUPLE CHEMOTHERAPY REGIME                      477

logical side effects are now a minor problem and in no patient was treatment
discontinued for this reason.

The most consistent response was seen in carcinoma of the breast, where all
patients responded in some degree. Regression was clinically complete in 6 out
of 14 cases. As all these patients had undergone previous hormonal or cytotoxic
treatment these results can be considered eiicouraging.

There were only 10 patients with head and neck cancer, but a remission in 8
cases compared well with the report of Leone et al. (1968), and considerably better
than the results of intra-arterial chemotherapy at this Centre.

Fourteen cases of carcinoma of bronchus were treated and 8 underwent brief
remission (3 months or less). It is recognised that it is difficult to palliate bronchial
carcinoma with cytotoxic agents and the use of quadruple chemotherapy in this
small series is not encouraging.

Surprisingly no remissions were achieved with carcinoma of the ovary cervix
or testicle.

The success claimed by Constanzi and Coltman (1969) was not confirmed in
this series of melanomas, and we no longer treat this tumour with the quadruple
regime.

In a small group of 6 soft tissue sarcomata only I failed to respond. If in a
larger series this response rate were to be maintained it would be an improvement
on previous experience (Newton, 1967; Wiltshaw, 1967).

In the small group of gastro-intestinal cancers, 4 out of 5 cases of carcinoma
of the large bowel and stomach responded, and it is felt that where 5-fluorouracil
has failed, quadruple chemotherapy may be of value.

TABLE XIII.-Response of Different Groups of Solid Tumours to the Modified

Quadruple Regime

Good remission           Moderate remission            No remission

Breast carcinoma      Head and neck carcinoma          Malignant melanoma

Soft tissue sarcoma              Ovarian carcinoma

Cervical carcinoma               Testicular carcinoma
Bronchial carcinoma              Bone sarcoma
Stomach and large bowel carcinoma

Table XIII summarises the value of this modified quadruple regime in the
different groups of solid tumours.

We would like to thank our colleagues who kindly referred their patients to us
and the junior medical and nursing staff for their care of the patients, Mrs.
Chatfield for her kind and patient assistance with the preparation of this paper,
Dr. Peter Hansell and the staff of the Medical Photographic department and Dr.
Peter Emerson for interpreting the lung function studies.

REFERENCES
BATEMAN, J. C.-(1962) J. Am. Geriat. Soc., 10, 721.

BUIELNS, B. C. ? JR., UNDERWOOD P. B., JR. ANDRUTLEDGE, F. N.-(1969) ' Cancer of the

!Uterus and Ovary'. Chicago (Year Book Medical Publishers Inc.) p. 123.
CONSTANZI, J. J. AND COLTMAN, F. J.-(1969) Cancer, N.Y., 23, 589.

HEIDELBERGER, C. AND ANSFIELD, F. N.-(1963) Cancer Res., 23, 1235.

478          I. W. F. ILA-NHAM, K. A. NEWTON AND G. WESTBURY

K.A?XOFSKY, D. A. AND BuiacHimNAL, J. H.-(1948) 'Evaluation of Chemotherapeutic

Agents.' Edited by Cohn M. MacLeod. New York (Columbia University
Press) p. 191.

Ku-NKLFm, P. B., Ev"s, I. H., JoNEs, V. AND WONG, K. K.-(1968)' Prognostic Factors

in Breast Cancer.  Edited by Forrest/Kunkler. London (E. & S. Livingstone
Ltd.) p. 213. '

LEONE, L. A., ALBAT,-A, M. M. AND RIMGE, V. B.-(1968) Cancer, N. Y., 21, 828.
NEwTON, K. A.-(1967) Br. J. Radiol., 40, 823.

WMTSIUW) E.-(1965) J. Obstet. Gynaec. Br. Commmw., 72, 590.-(1967) Br. med. J.,

ii) 142.

				


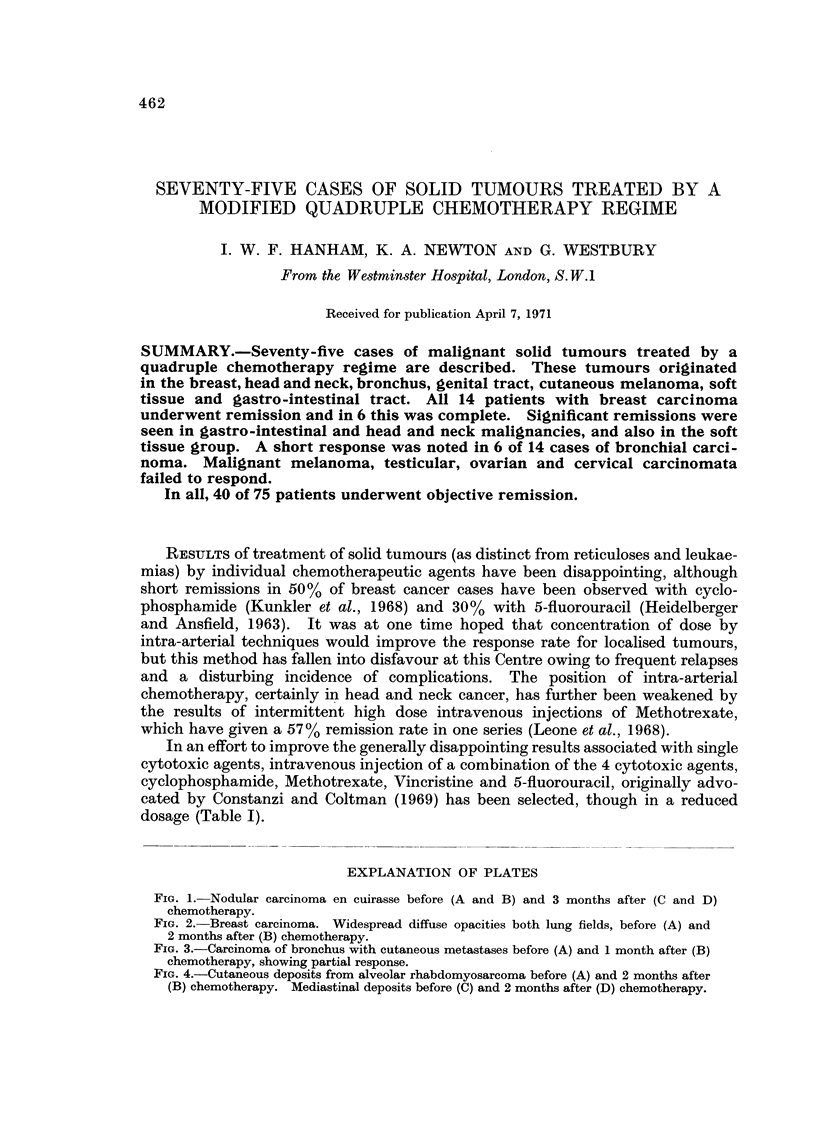

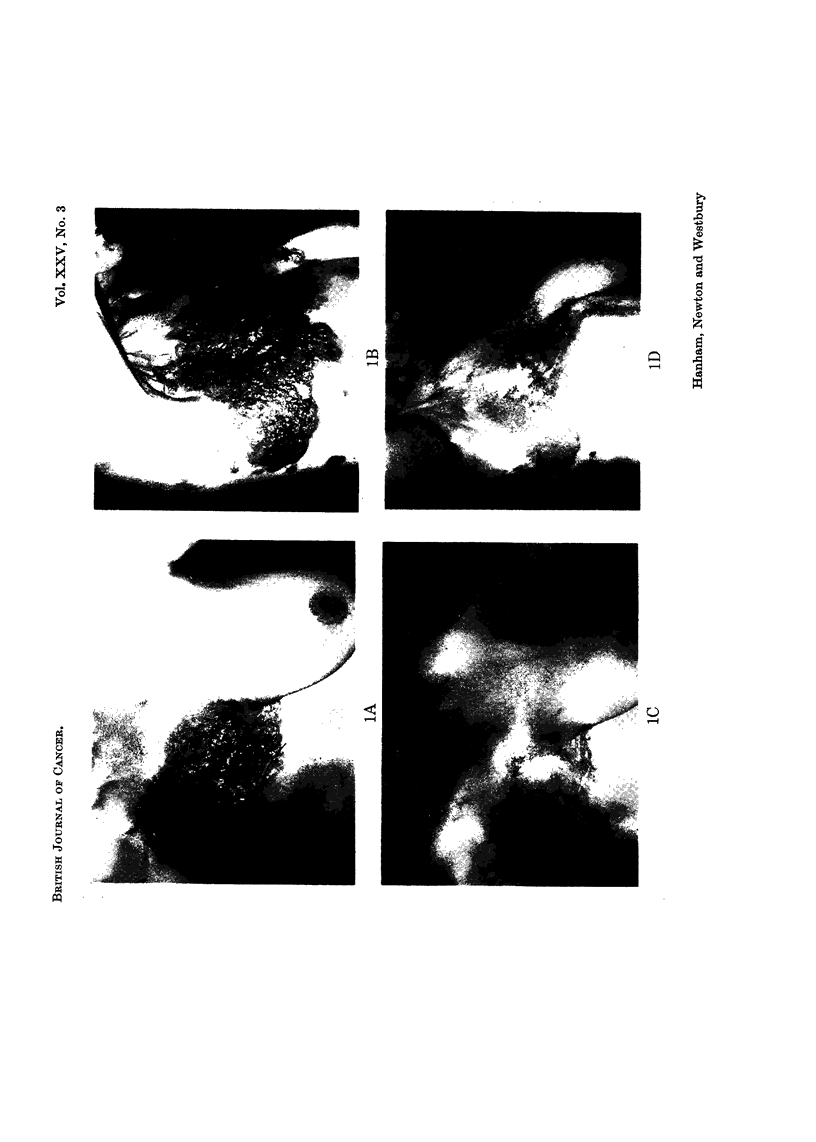

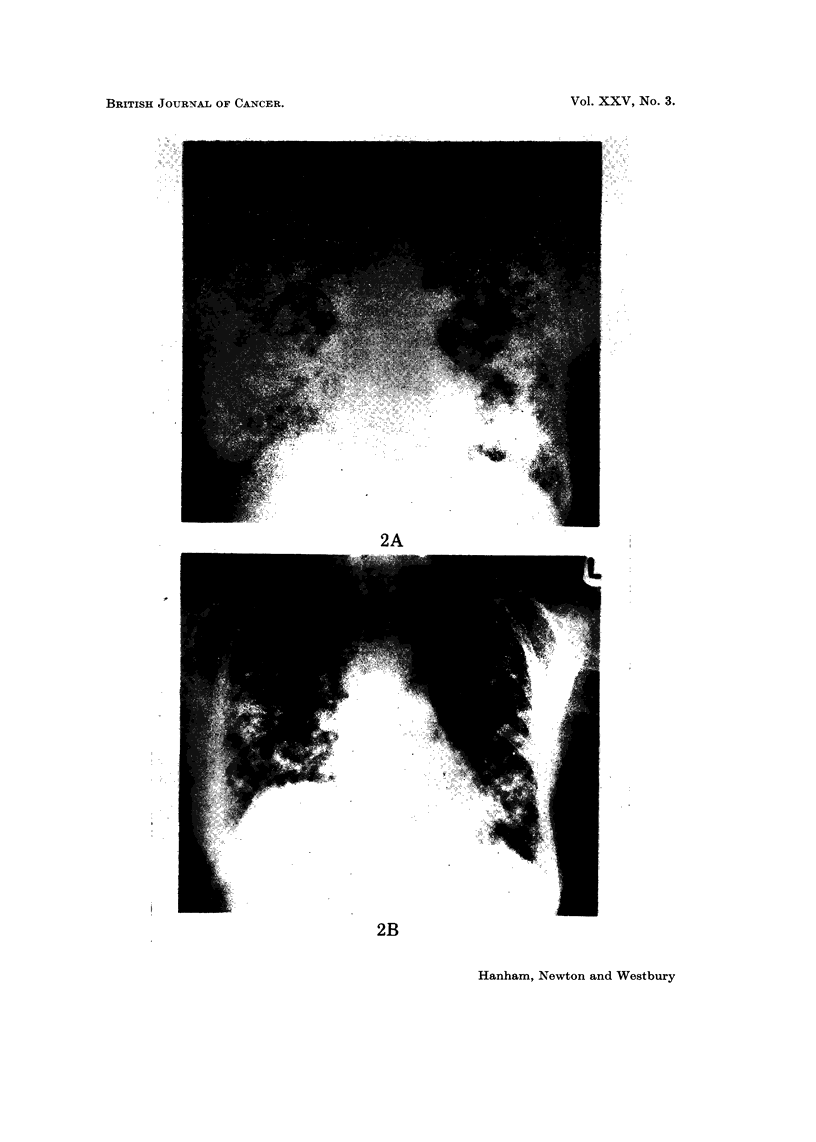

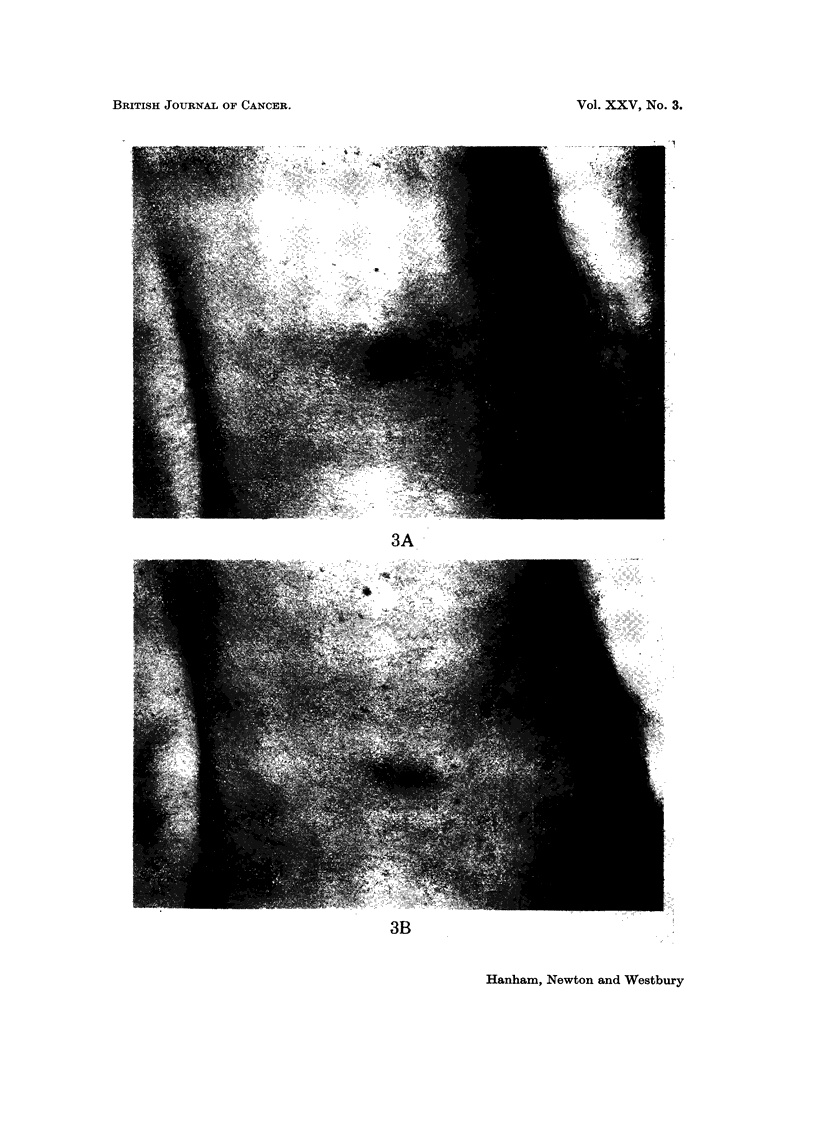

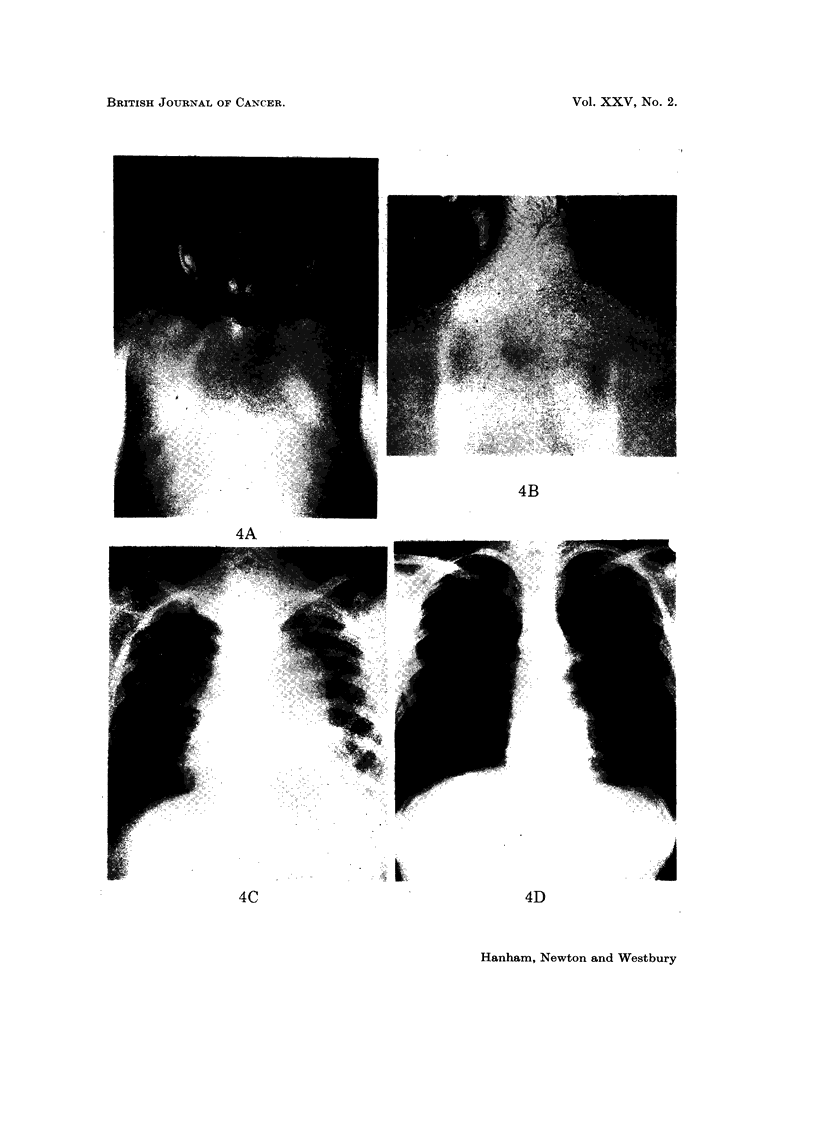

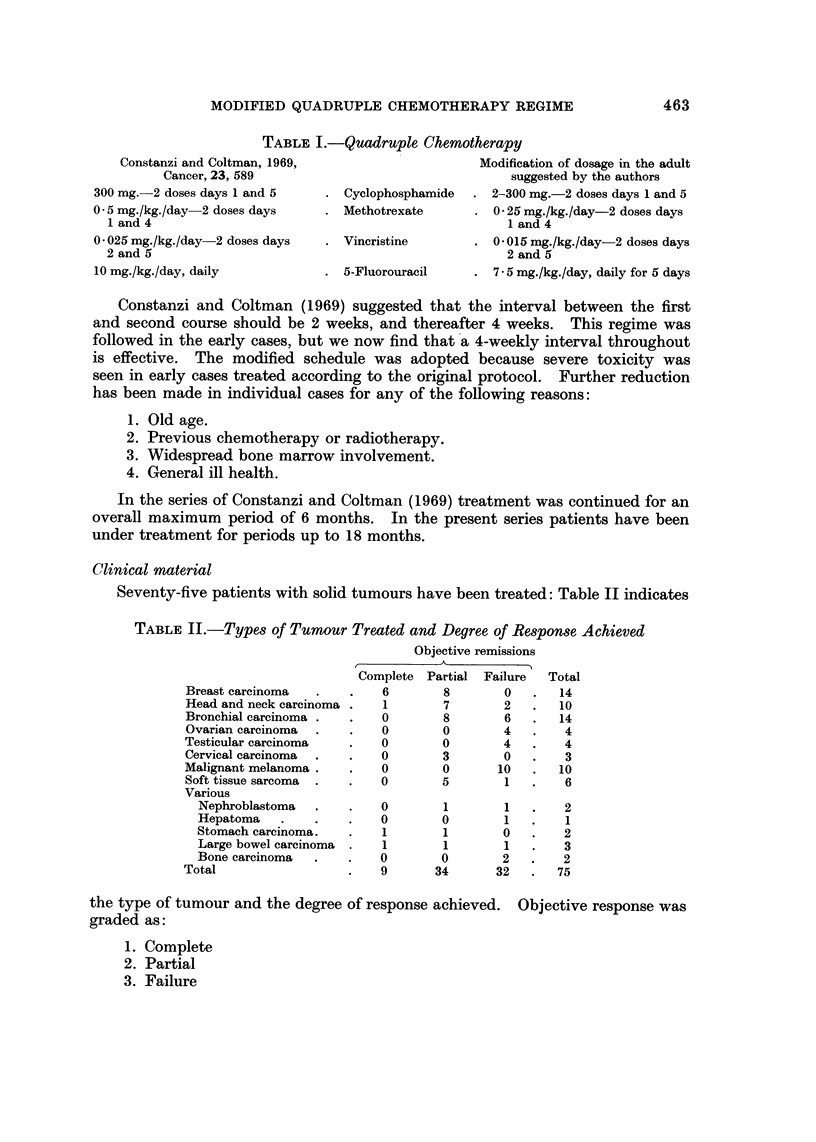

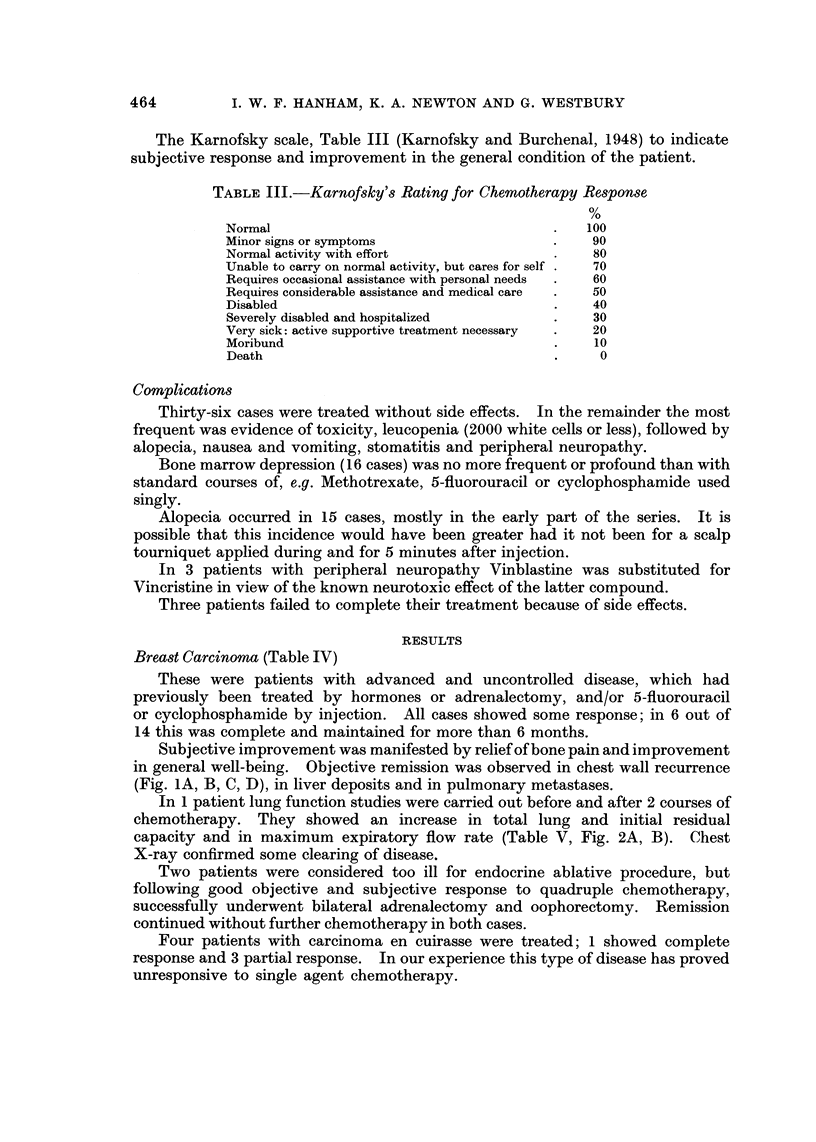

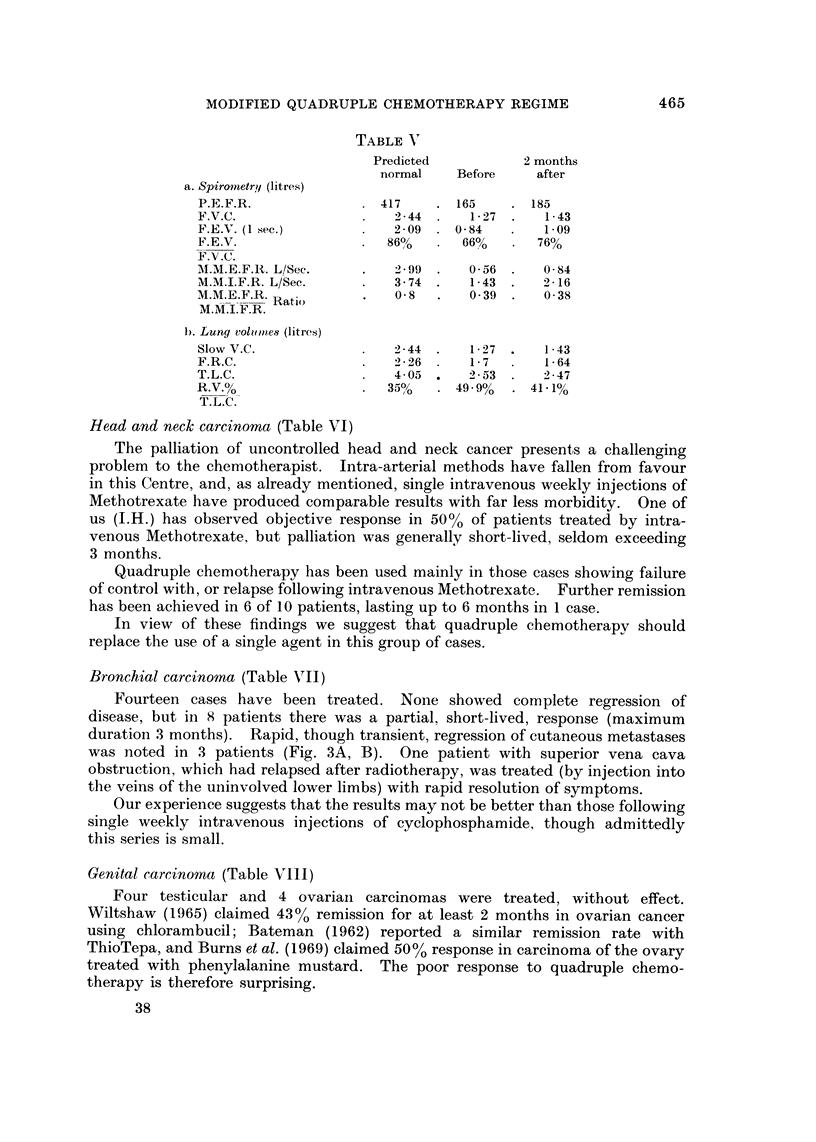

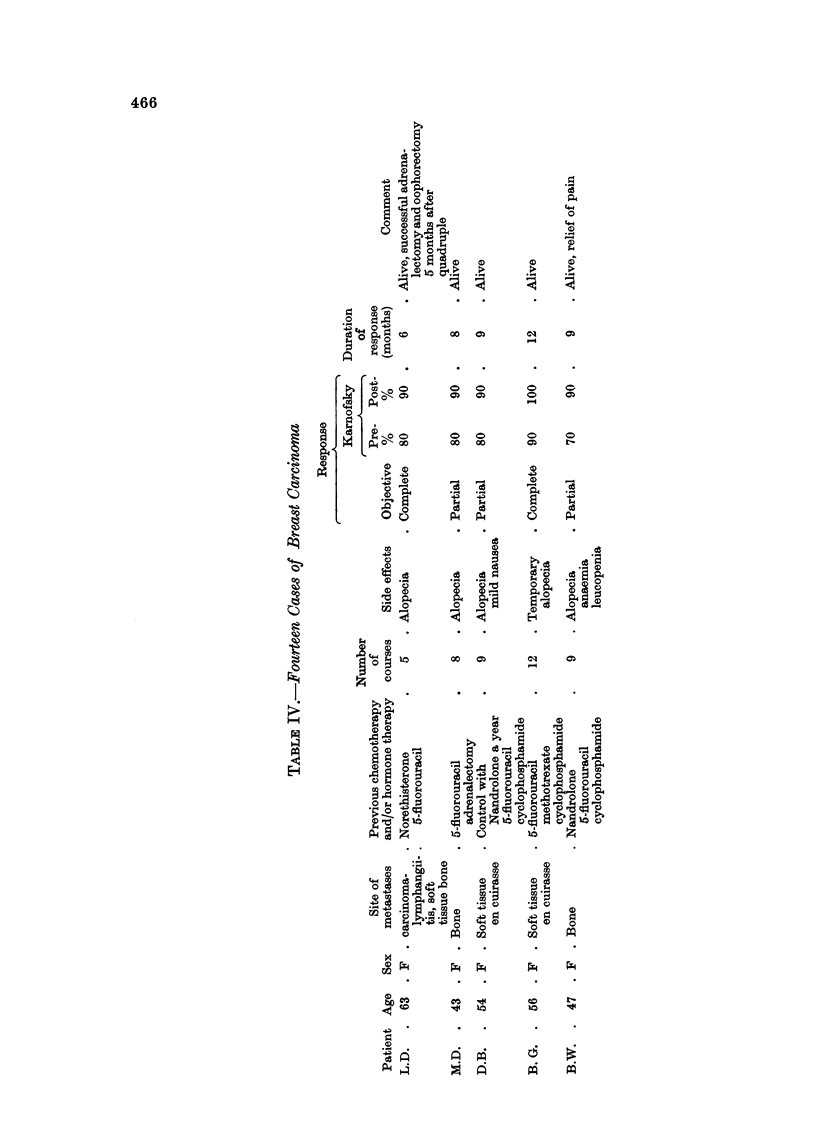

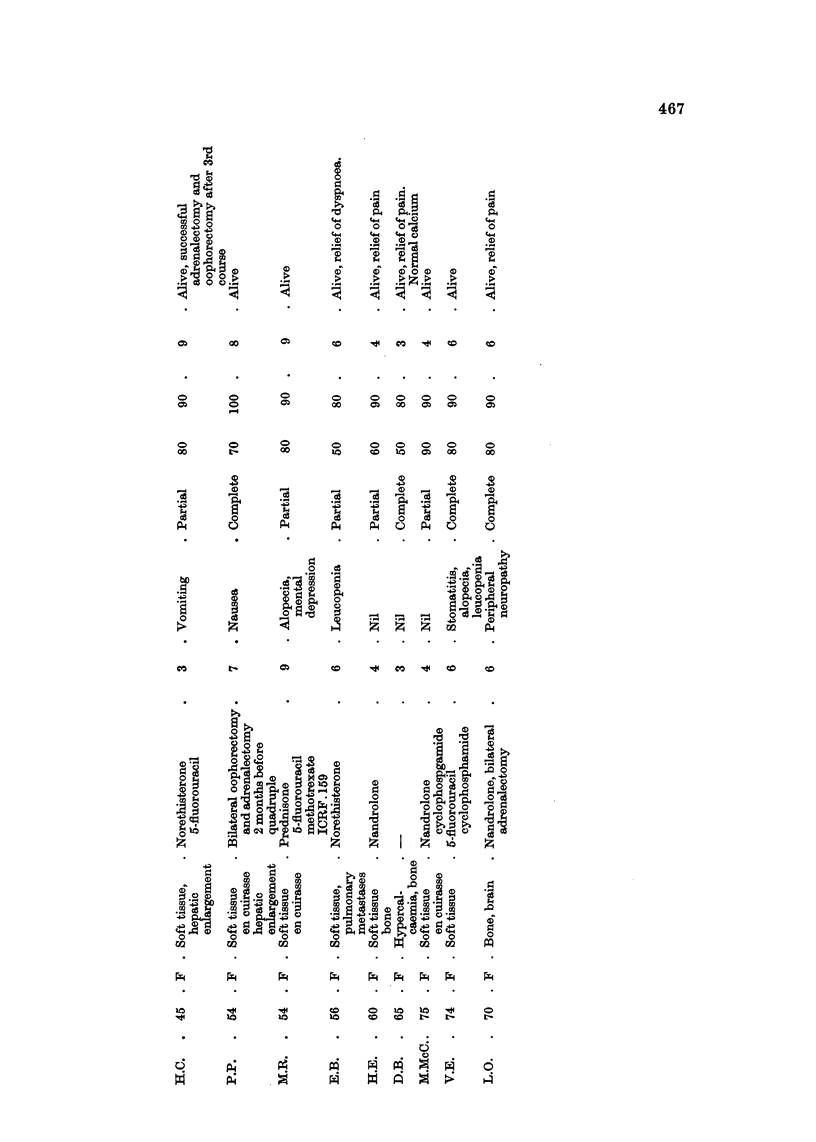

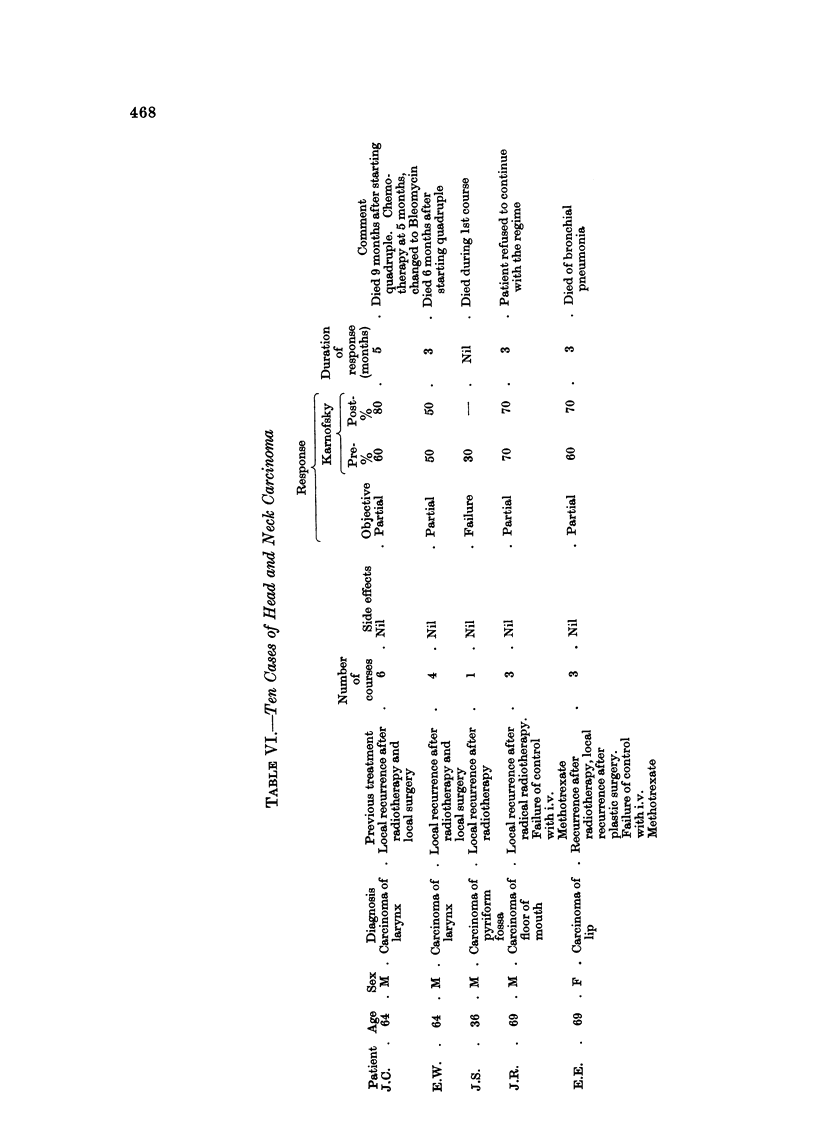

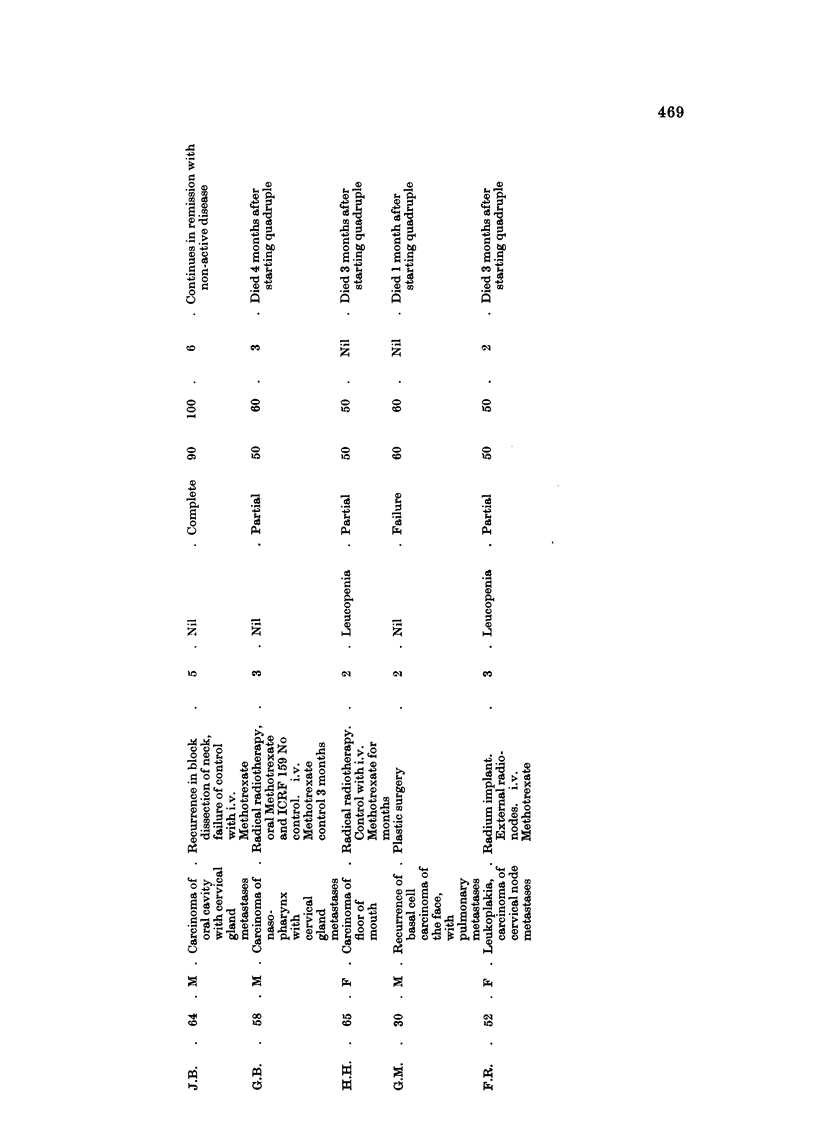

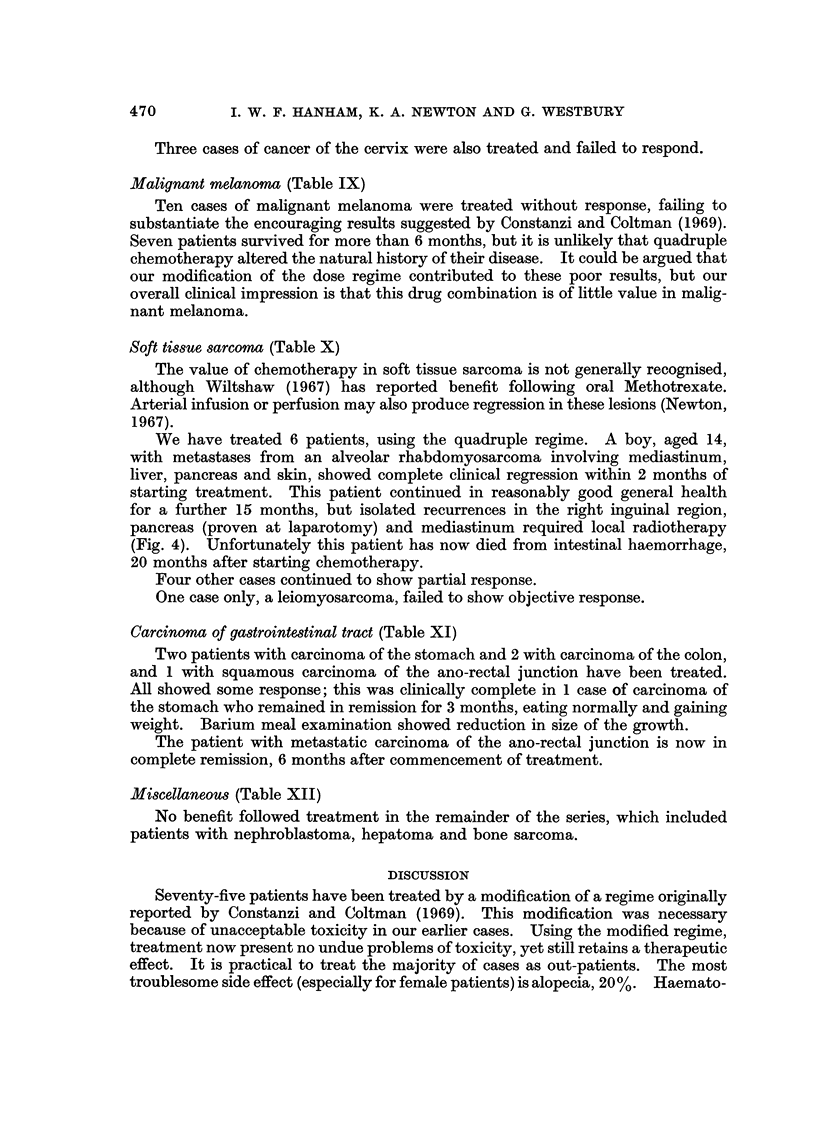

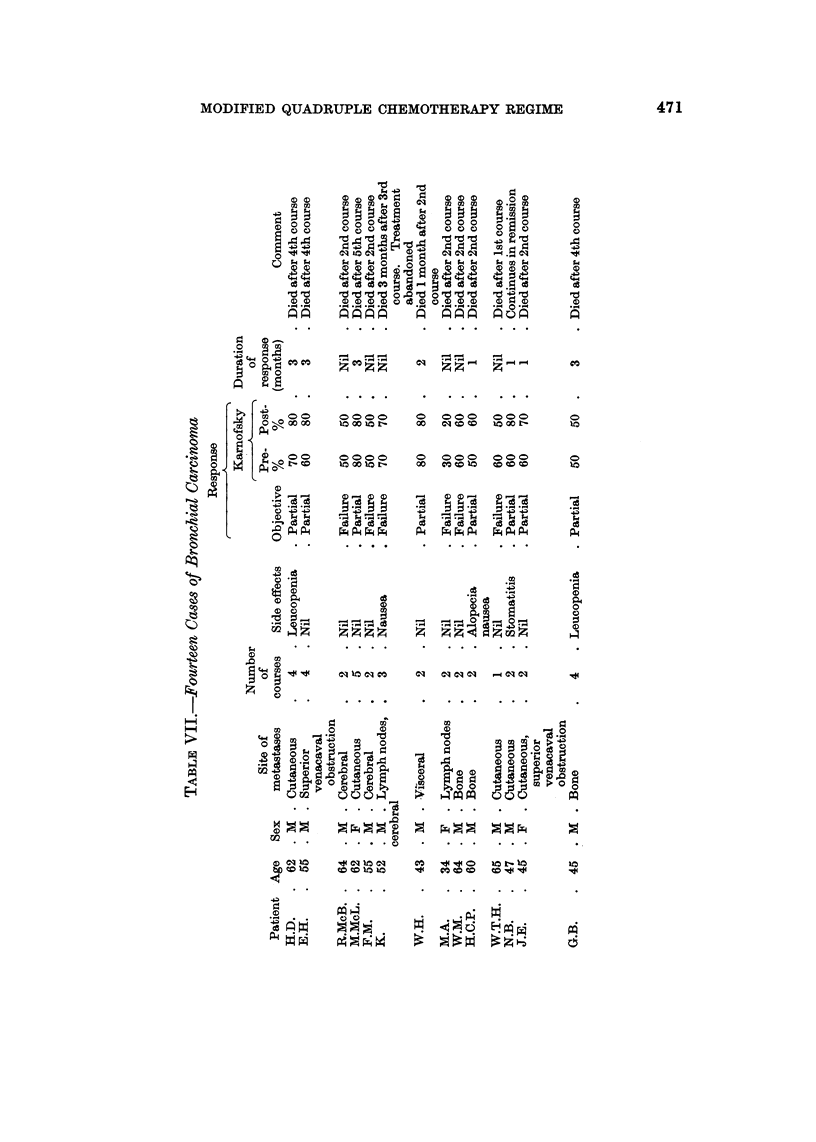

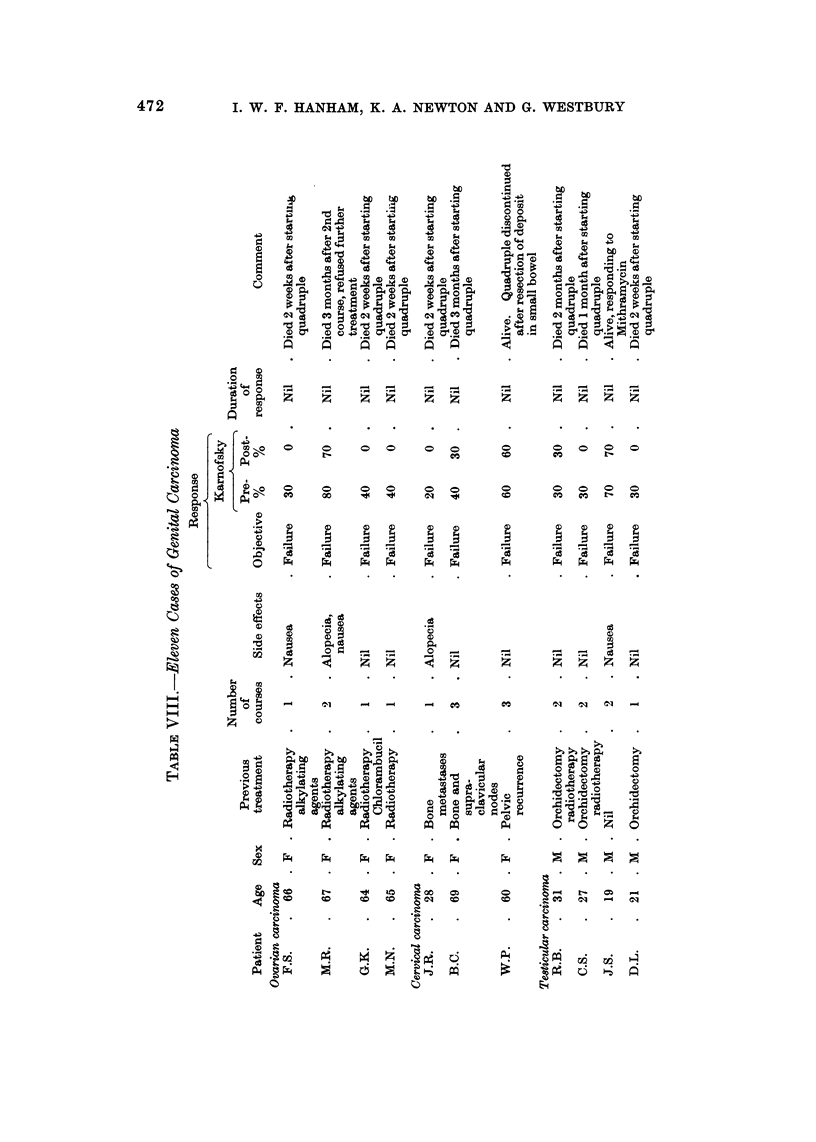

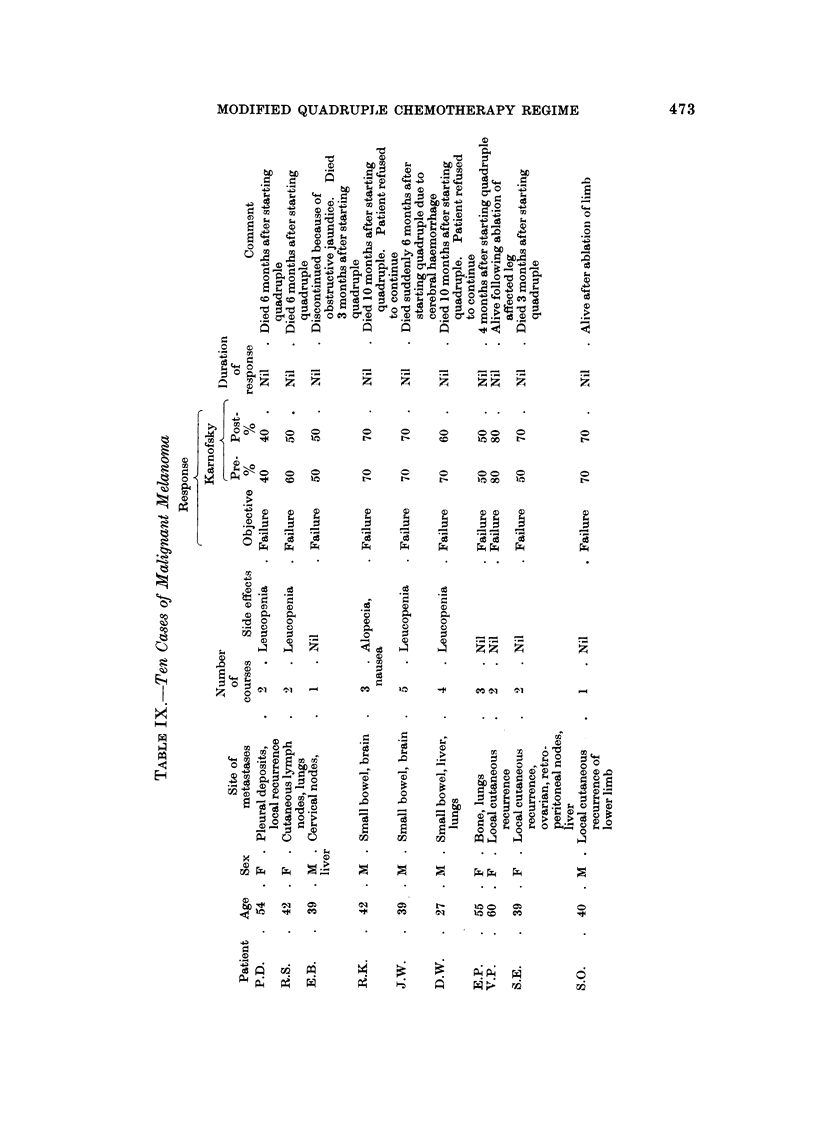

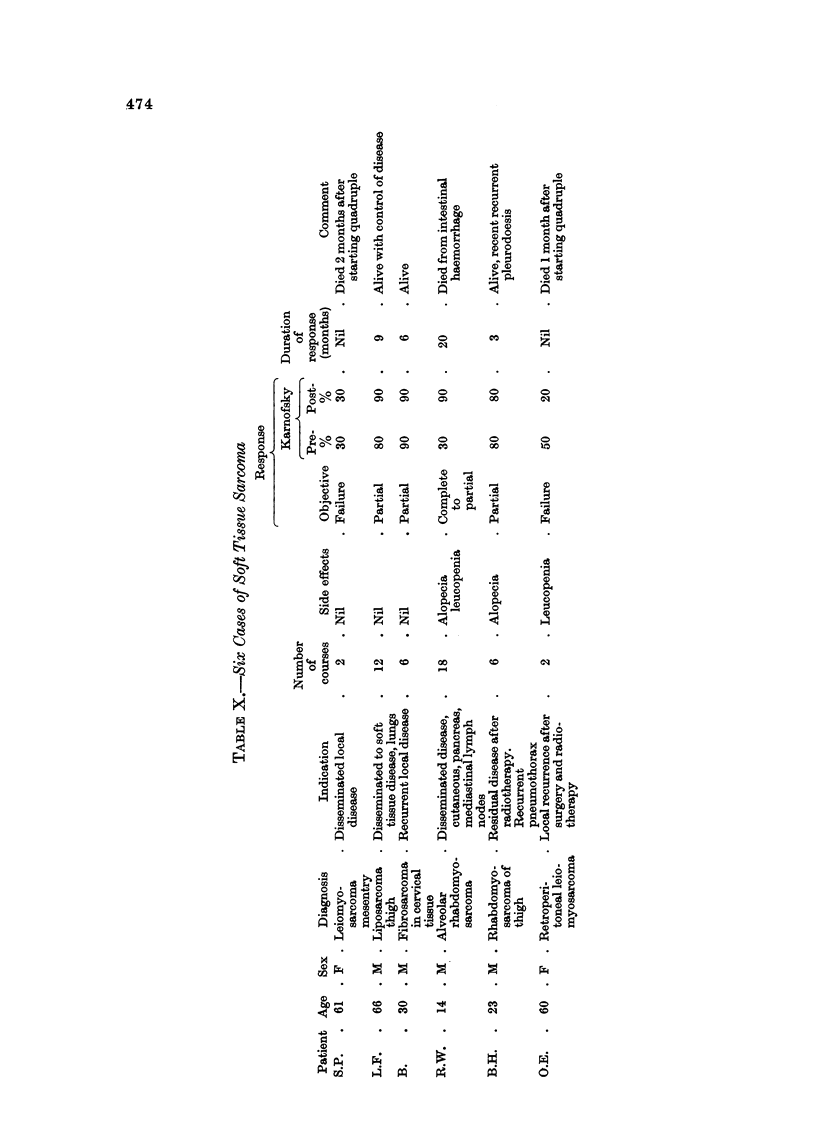

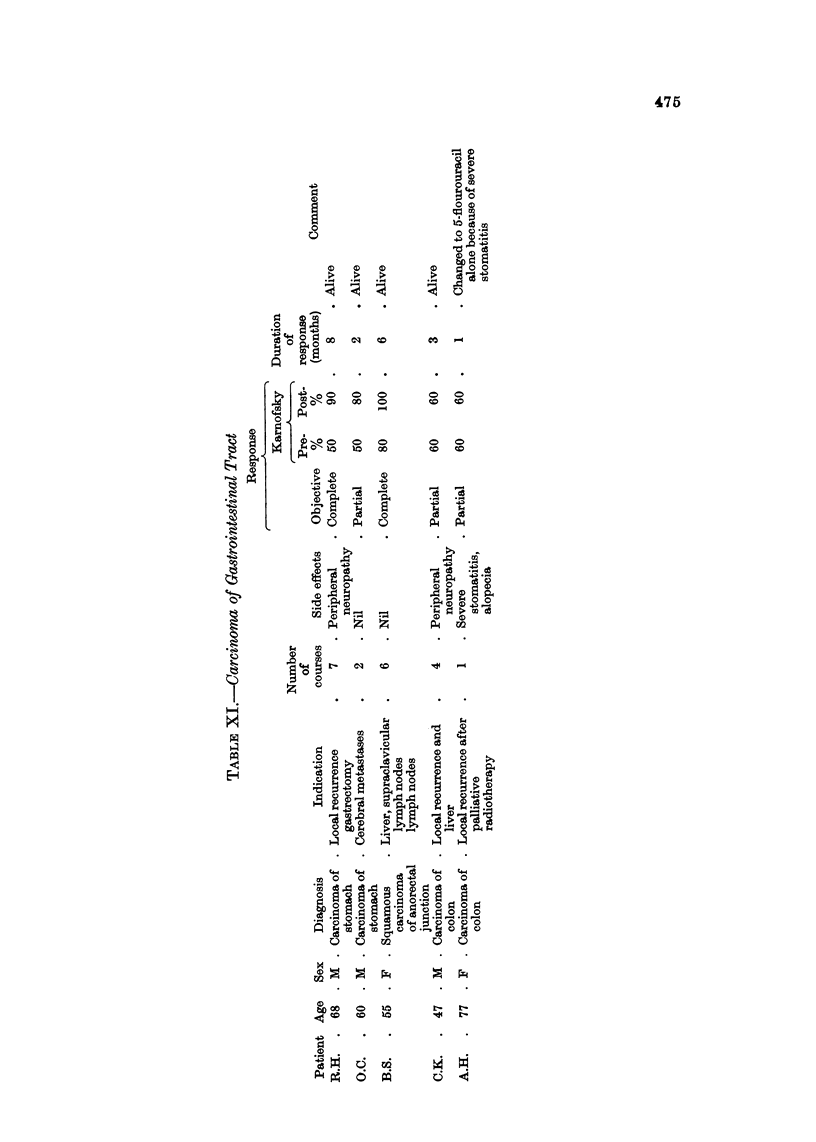

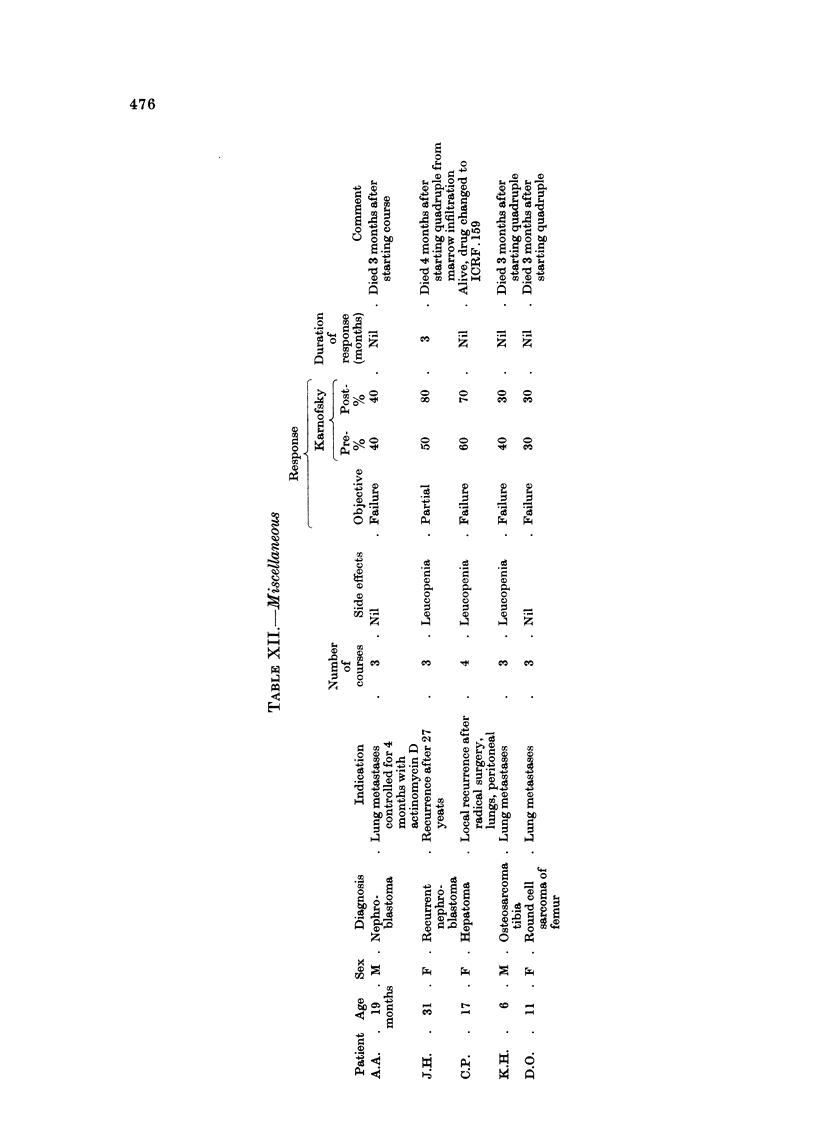

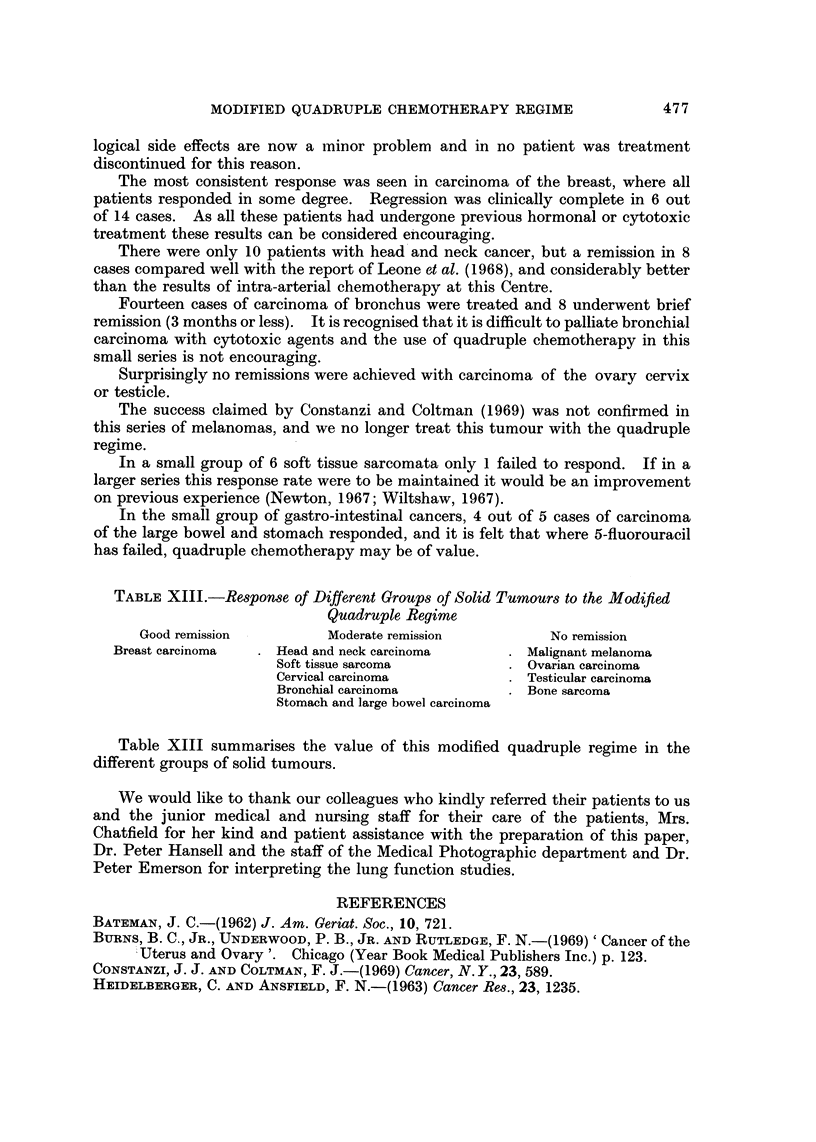

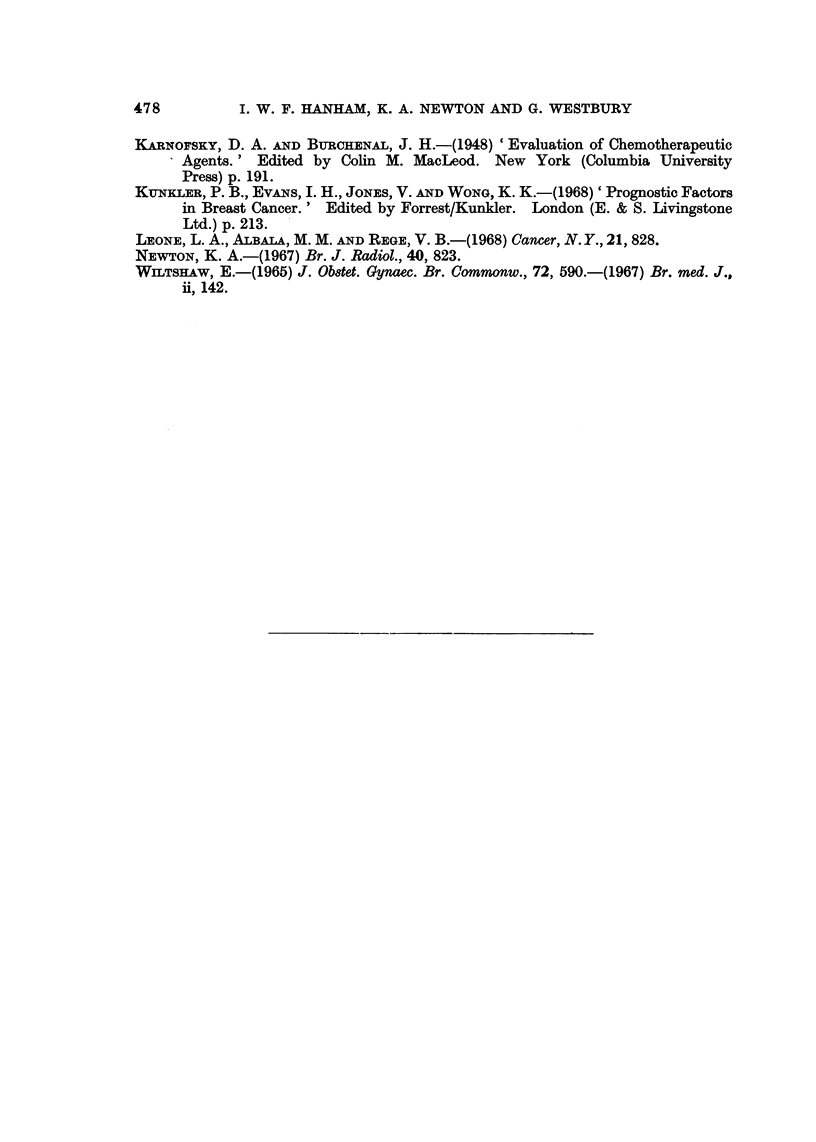

